# Downregulation of Peroxidase Activity of Platinum Cube Enables Minute–Time Scale Colorimetric Signaling of Hypoxanthine for Fish Freshness Monitoring

**DOI:** 10.3390/foods12020291

**Published:** 2023-01-08

**Authors:** Xiaoming Ma, Tingting Feng, Peng Zhang, Hui Zhang, Xuan Hu, Yuying Yang, Zhen Wang, Huifang Zhang, Dong Peng, Xun Li, Jianguo Xu

**Affiliations:** 1 Key Laboratory of Organo-Pharmaceutical Chemistry of Jiangxi Province, School of Chemistry and Chemical Engineering, Gannan Normal University, Ganzhou 341000, China; 2 Fujian Province-Indonesia Marine Food Joint Research and Development Center, Fujian Polytechnic Normal Univeristy, Fuzhou 350300, China; 3 School of Food and Biological Engineering, Hefei University of Technology, Hefei 230009, China

**Keywords:** food safety, nanozyme, fish freshness, platinum nanocube, hypoxanthine

## Abstract

Due to its unique biological composition, aquatic products, especially fish, are extremely perishable compared to other muscle products. Herein, we proposed an artificial nanozyme-based colorimetric detection of hypoxanthine (Hx), the indicator of fish freshness, in a minute–time scale without the assistance of a natural enzyme (hypoxanthine oxidase). The principle is based on the interaction between Hx and polyvinylpyrrolidone-modified platinum cubic nanomaterials (PVP-PtNC), in which the catalytic active sites of PVP-PtNC’s surface were blocked by Hx. This causes the downregulation of PVP-PtNC’s catalytic ability and weakened its ability to catalyze the oxidization of 3,3′,5,5′-Tetramethylbenzidine (TMB) by H_2_O_2_. Accordingly, the decrease in the UV–vis absorption and the weakening of the colorimetric reaction color is proportional to the Hx concentration. On this basis, a target-triggered colorimetric method for detecting Hx is developed for fish freshness monitoring with a fast detection speed, low cost, high accuracy, and simplified operation. Experiments reveal that the correlation response of Hx is from 0.5 μM to 10 mM with a limit of detection of 0.16 μM. In particular, the Hx detected from real fish indicates that the method possesses a promising potential for practical application. All of these features are expected to promote the development of online detection tools for food safety monitoring.

## 1. Introduction

As one of the most important components of the modern food industry, aquatic products are especially favored by humans because they not only offer essential calories to the human body but also provide multiple nutrients such as protein and docosahexaenoic acid [1]. According to the official report by fishery and aquaculture products fishery and aquaculture products (FAPs), the consumption of aquatic products is increasing steadily in people’s daily diets [2]. China has ranked first in fisheries and aquatic products in the world [3], most of which is consumed by the domestic market and China is responsible for most of the increase in world fish consumption. However, aquatic products, especially fish, are highly perishable with a limited shelf-life after slaughter due to the ready occurrence of protein degradation, lipid oxidation, and changes in odor, flavor, and texture [4,5]. The aquatic product spoilage thus stimulates the accurate freshness analysis of fish. Traditionally, consumers mainly rely on a sensory approach to judge the organoleptic features of fish according to their perceptions and experiences, which is not reliable and is subjective for freshness evaluation and might even cause severe food safety incidents if the food had been spoiled for a long time. Therefore, it is imperative to develop accurate analytical methods for accurately monitoring aquatic products’ freshness.

With the rapid development of aquatic product science and the advancing of modern analytical chemistry, the methods adopted for aquatic product freshness, exemplified by fish, identification has been at the biochemical and microbiological level. For instance, the growth of microorganisms in fish tissues leads to the degradation of adenosine triphosphate (ATP) but the right accumulation of a reaction intermedium of hypoxanthine (Hx) [6,7]. This transform means that Hx can be used as an important indicator to reveal the freshness of fish since its accumulation is proportional to the storage time. Currently, to meet the freshness standard, conventional methods including mass spectrometry technology [8,9] near-infrared spectra [10], electronic nose [11], electronic tongue technology [12], fluorescence [13,14,15], computer vision technology [13,16], and gas chromatography–mass spectrometry (GC–MS) technology [15] have been already been applied to realize the measuring of Hx. Although these methods are highly repetitive, accurate, and reliable, the request for expensive equipment, professional personnel, and specialized laboratory limits their wide application. The ask for a user-friendly detection method with fast detection speed, low cost, high accuracy, and simplified operation is continually demanded.

In the past decade, nanozymes, as a new generation of artificial enzymes with highly effective enzyme-like properties, come into focus and achieve great progress. The landmark work is reported by Yan’s group in 2007. In their work, they first found the intrinsic peroxidase-like activity of ferromagnetic nanoparticles [17]. From that time, several nanozymes (e.g., Au nanoclusters [18,19], WS_2_ nanosheets [20], graphene oxide [21], and carbon dots [22]) have been explored and applied for building biosensors in responses to a variety of target analytes including proteins, nucleic acids, metal ions, inorganic and organic compounds, and so on [23,24,25,26,27]. In particular, noble metal nanomaterials have become one of the hot topics due to their unique chemical properties and excellent catalytic activity. Moreover, compared with natural enzymes that are difficult to prepare, easy to deactivate, and expensive to use, artificial nanozymes can be easily prepared with low cost, high stability, and especially, high catalytic ability [26]. These advantages allow the boom of nanozyme-based sensors for biochemical analysis, which might also be useful for us to build a Hx-responsive online detection method with high assay performance.

The conventional Hx sensing platform typically requires xanthine oxidase (XOD) to convert Hx to H_2_O_2_ and uric acid in the presence of oxygen. The generated H_2_O_2_ can be further catalyzed and produce hydroxyl radicals (•OH) via the catalysis of peroxidase-mimicking nanozyme and oxidize the chromogenic substrate from colorless to colored [28,29]. For instance, Zhang et al. demonstrated an efficient Hx-sensing platform based on the peroxidase-mimicking activity of Fe-doped polydopamine (Fe-PDA) [30]. In the presence of Hx, XOD can quantitatively convert Hx to H_2_O_2_, and the generated H_2_O_2_ can further oxidize the colorless 3,3′,5,5′-Tetramethylbenzidine (TMB) to blue oxTMB with the catalysis of Fe-PDA. However, these biosensors still need the assistance of the natural enzyme (XOD), which meant it was difficult to avoid the drawbacks of conventional enzymes such as their instabilities under harsh conditions.

Taking the above into consideration, the research objective is to construct a conventional colorimetric sensing platform for fish freshness determination. Herein, we hypothesized a colorimetric method for the determination of Hx based on the downregulation of peroxidase activity of the polyvinylpyrrolidone-modified platinum cubic nanomaterials (PVP-PtNC). This strategy lies in our finding that the Hx can block the active catalytic sites on the PVP-PtNC surface so that the ultra-high catalytic ability of PVP-PtNC can be downregulated directly by Hx. Once encountered with the H_2_O_2_ to catalyze the oxidation of TMB, a typical peroxidase substrate, the significantly weakened peroxidase activity would powerfully inhibit the generation of oxidized blue products. Therefore, this sensing platform can realize the quantitative determination of Hx without the assistance of a natural enzyme (XOD). Our results showed that the morphology of PVP-PtNC was well demonstrated by transmission electron microscopy (TEM). The decrease in the UV–vis absorption and the weakening of the colorimetric reaction color is proportional to the Hx concentration. Moreover, benefiting from the wide linear response range, low detection limit, high specificity, and repeatability, this colorimetric nanosensing method has been successfully applied to analyze real fish samples in only 10 min. Such a nanozyme-based sensing platform with its minute-time scale detection merit would thus open a new avenue for the online monitoring of aquatic products’ freshness.

## 2. Materials and Methods

### 2.1. Materials and Instruments

Hypoxanthine (Hx), uric acid, dopamine, and 3,3′,5,5′-tetramethylbenzidine hydrochloride (TMB) were purchased from Shanghai bioengineering technology co. LTD. (Shanghai, China). Hydrochloric acid (HCl), acetic acid (CH_3_COOH), sodium acetate (CH_3_COONa), sodium hydroxide (NaOH), Glucose (Glucose), hydrogen peroxide (H_2_O_2_, 30 wt%), ascorbic acid (C_6_H_8_O_6_), zinc chloride (ZnCl_2_), magnesium chloride (MgCl_2_), Calcium chloride (CaCl_2_), silver nitrate (AgNO_3_), potassium chloroplatinate hexahydrate (Ⅳ) (K_2_PtCl_6_·6H_2_O), potassium bromide (KBr), polyethylene pyrrolidone (PVP, MW ≈ 50,000), and ethylene glycol (EG) were purchased from Sigma Aldrich (St. Louis, MO, USA). Live fish were purchased from the supermarket located in the Gannan Normal University (Jiangxi, China). All reagents were used directly without further purification.

The UV–vis spectrophotometer (UV-1780, Shimadzu, Kyoto, Japan) was used to record UV–vis absorption. The Precision Electronic Balance (BSA2245, Sartorius, Goettingen, Germany) was used to weigh reagents. Transmission electron microscope (Titan G260-300, FEI Company, Hillsboro, OR, USA) was used to characterize the morphology of nanoparticles. The concentration of Platinum element was determined with the inductively coupled plasma emission spectrometry (ICP-OES) (Ultima2, HORIBA Jobin Yvon, Longjumeau, France). The High-Speed refrigerated centrifuge (Sorvall ST 16R, Thermo Scientific, Waltham, MA, USA) was used to separate and enrich samples. The water preparation apparatus (Milli-Q, Millipore Corporation, Bedford, MA, USA) was used to prepare ultrapure water.

### 2.2. Synthesis of PVP-PtNC

The PVP-PtNC nanomaterials were synthesized according to literature reports [31]. First, 20 mg KBr and 40 mg PVP were added into a 3.5 mL glycol (EG) solution. After dissolving, the mixture was heated to reflux (about 180 °C) for 15 min under an oil bath. This is followed by adding 0.5 mL K_2_PtCl_6_·6H_2_O solution (40 mg/mL, EG dissolved) to react with the mixture for another 20 min. Subsequently, the resultant solution was immediately cooled with an ice bath. The excess PVP was removed by centrifugation and the final reaction products were washed with acetone and deionized water 3 times to obtain the purified PVP-PtNC, which was stored in 4 mL ultrapure water at 4 °C before usage. The concentration of platinum was measured to be 0.473 g/L in PVP-PtNC by ICP-OES.

### 2.3. PVP-PtNC-Based Colorimetric Sensing of Hx

Prior to detecting Hx, the standard Hx solutions were prepared by weighing 0.0136 g Hx and dissolving it in 0.4 mL of 1 M HCl since Hx is poorly soluble in neutral conditions. The resultant mixture was then added with 9.6 mL of 0.05 M Sodium acetate-Acetic acid (NaAC-HAC) buffer (pH = 4) to obtain the storing solution of Hx (10 mM). Other concentrations of Hx were obtained by diluting the storing solution of Hx with 0.05 M NaAC-HAC buffer (pH = 4). To conduct the colorimetric detection of Hx, the PVP-PtNC (100 μL, 5 μM) and Hx solutions at different concentrations were mixed in 0.05 M NaAC-HAC buffer (pH = 4) and incubated at room temperature for 30 min. The resultant mixture (100 μL) was added with H_2_O_2_ (25 μL, 1 M) and TMB (25 μL, 15 mM) to carry out the colorimetric catalysis reaction at room temperature for 10 min. The visible absorption spectra from 400 to 800 nm were measured by a UV–vis spectrophotometer and the peak absorption value at 651 nm was recorded for evaluating its sensing performance.

### 2.4. Processing of Real Fish Samples

Firstly, we took fresh live fish and removed their scales, skin, and bones to obtain the fresh fish meat, which is then processed into minced meat by grinding treatment and spoilage-treated by placing them at room temperature. After 12 h, 5.0 g minced fish meat was added with H_2_SO_4_ (10 mL, 0.06 M) solution to make them digested, and then added with 39 mL of ultrapure water to break up the fine tissue by homogenate processing. The resultant solution was placed in a shaker for 4 h at room temperature. After the shaking was completed, the residue was filtered with filter paper, and the maintained solution phase was filtered twice with an organic filter followed by adding NaOH (1 mL, 6 M) to adjust its pH to 4.0. The final sample was placed in the refrigerator and stored at 4 °C until use.

## 3. Results and Discussion

### 3.1. Principle of the Proposed Colorimetric Sensor

**Figure 1:**
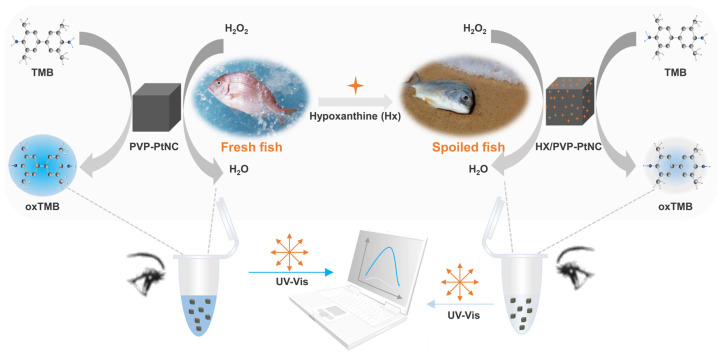
Schematic diagram of the working principle for the PVP-PtNC-based colorimetric detection of Hx.

Figure 1 presents the working diagram of the PVP-PtNC-based colorimetric sensor to detect Hx. One can find that, in the absence of Hx, the PVP-PtNC can maximally maintain its peroxidase activity. When exposed to the mixture of H_2_O_2_ and TMB, the oxidization of TMB by H_2_O_2_ can be immediately performed by the catalytic peroxidase activity of PVP-PtNC, in turn resulting in an obvious solution color change and a significant enhancement of the UV–vis absorption for the Hx-absent negative sample. However, in the presence of Hx, the surface with catalytic active sites on PVP-PtNC can be coated with Hx because of the physical absorption of Hx on PVP-PtNC. The catalytic activity of PVP-PtNC is thus downregulated with the net decrease in its active sites. Upon being introduced with H_2_O_2_ and TMB, the oxidization of TMB by H_2_O_2_ cannot be strongly executed anymore. We can only observe a weakened color change or even a colorless solution of the Hx-presented positive sample. In this case, the signal absorption is also decreased. From the working principle, it is seen that the color change degree and the decrease in the UV–vis absorption are based on the Hx concentration since the more the Hx presented, the more active sites of the PVP-PtNC will be occupied. The color change can be easily observed by the naked eye, while the intensity change of UV–vis can be monitored by a UV–vis spectrometer.

### 3.2. Feasibility Demonstration for Hx Analysis

**Figure 2:**
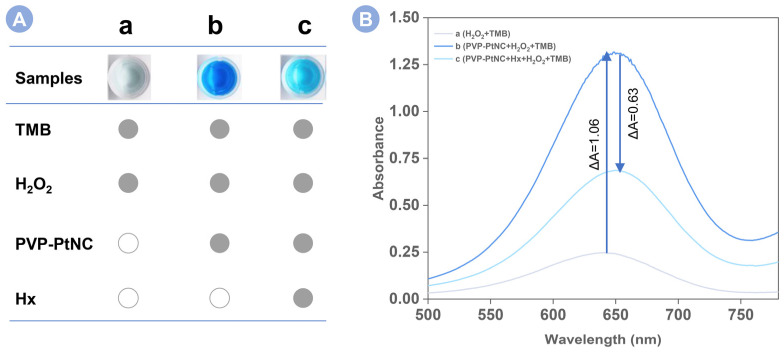
(**A**) Photographed images and (**B**) UV–vis absorption spectra of (a) TMB + H_2_O_2_, (b) PVP-PtNC + TMB + H_2_O_2_, and (c) PVP-PtNC + Hx + TMB + H_2_O_2_.

To validate the feasibility of Hx analysis, the strong peroxidase activity of the platinum cube is the prerequisite for subsequent studies, which should be confirmed first. As shown in sample a of Figure 2A, the mixture of TMB and H_2_O_2_ showed a very weak color, suggesting that the autocatalysis of TMB by H_2_O_2_ is very difficult. After added with PVP-PtNC, as shown in sample b, we can excitingly find that the solution was changed to dark blue. This phenomenon confirms the strong peroxidase activity of PVP-PtNC. On this basis, we tested the catalysis system for Hx sensing in sample c. As expected, the pre-incubation of Hx with PVP-PtNC, and then, using the Hx-coated PVP-PtNC to react with the mixture of TMB and H_2_O_2_ was unable to strongly catalyze the oxidization of TMB by H_2_O_2_ since sample c only showed a light blue. The big difference in samples b and c demonstrate the downregulation of the peroxidase activity of PVP-PtNC by Hx, which also evidences the availability of this sensing method for Hx analysis. We also further measured the UV–vis absorption spectra of samples a, b, and c in Figure 2B. It can be seen that the oxidization of TMB by H_2_O_2_ displayed the weakest UV–vis absorption in line a. The further introduction of PVP-PtNC caused a significantly improved peak absorption intensity in line b. However, when used to analyze Hx, the peak absorption was obviously decreased in line c. The variation tendency of the UV–vis absorption spectra in Figure 2B is consistent with the color changes in Figure 2A, confirming further the feasibility of Hx detection.

### 3.3. Characterization of as-Synthesized PVP-PtNC

In this study, the successful colorimetric sensing of Hx is based on the application of PVP-PtNC. We, therefore, need to first characterize the as-synthesized PVP-PtNC. As shown in Figure S1A, the synthesized PVP-PtNC were all cube-shaped and uniformly dispersed. The average particle size is about 7.3 nm. Figure S1B is a high-resolution TEM image of a single PVP-PtNC nanomaterial, which clearly showed the corresponding lattice fringe and the lattice fringe spacing. These results indicate that PVP-PtNC has been successfully prepared.

The Michaelis constant (*K*m) value is a key indicator of enzymatic efficiency that indicates the affinity between enzymes and substrates, in which the lower *K*m value signifies a higher affinity [26]. To determine the peroxidase-like activity of the PVP-PtNC, a steady-state kinetic assay was carried out at room temperature. The Michaelis–Menten curves were obtained and fitted to the double-reciprocal plot (Figure S2A,B) and the *K*m of the PVP-PtNC toward TMB was calculated to be 2.22 × 10^−5^ M. The *K*m of the PVP-PtNC toward TMB was lower than that of HRP, suggesting that PtNC has a higher binding affinity to TMB compared with HRP (Table S1). The *K*m of the PVP-PtNC toward H_2_O_2_ was calculated by plotting the initial reaction velocities against the H_2_O_2_ concentration curve (Figure S2C) and then fitted to the double-reciprocal plot (Figure S2D). The Km was 3.92 × 10^−3^ M, indicating that PtNC had a higher binding affinity to H_2_O_2_ compared with HRP. Therefore, PtNC was a promising peroxidase candidate for the peroxidase-like enzymatic reaction.

### 3.4. Exploring the Interaction of PVP-PtNC with Hx

**Figure 3:**
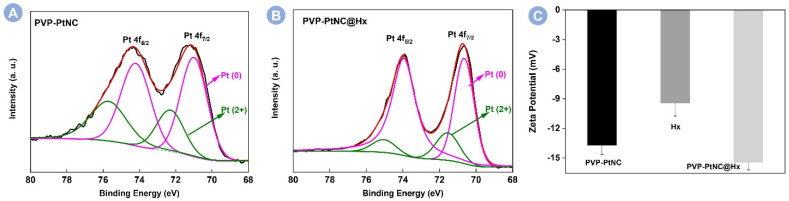
Pt 4f XPS spectra of PVP-PtNC before (**A**) and after (**B**) incubated with Hx. Black, red, magenta, and green lines represent the raw curve, the fitted curve, the Pt (0), and the Pt (II) components’ curves, respectively. (**C**) Zeta potential measurements of PVP-PtNC, Hx, and their mixture.

To deeply understand the interaction between PVP-PtNC and Hx, the charge states of Pt species are investigated by XPS. As seen in Figure 3A, the PVP-PtNC showed four peaks at 71.03, 72.33, 74.26, and 75.74 eV. According to the literature, the binding energies for Pt (0) 4f_7/2_ and 4f_5/2_ are 71.1 and 74.4 eV, respectively [32,33]. The peaks of PVP-PtNC at 71.03 and 75.74 eV can be assigned to Pt (0) 4f_7/2_ and 4f_5/2_, respectively. In the absence of target Hx, the fraction of Pt (II) and Pt (0) is determined as 34.6% and 65.4%, respectively. Interestingly, the addition of Hx to react with PVP-PtNC decreased the content of Pt (II) because the fraction of Pt (II) and Pt (0) is determined as 83.9% and 16.1% in Figure 3B. The comparative results indicate the reduction of Pt (II) by Hx. In addition, the zeta potential of the synthesized PVP-PtNC and Hx solution was −13.7 mV and −9.4 mV, respectively, and their mixture changed the zeta potential to –15.4 mV in Figure 3C. Therefore, Hx potentially regulates the peroxidase-like activity of PVP-PtNC through electrostatic adsorption and the in situ reduction interaction between Hx and PVP-PtNC.

### 3.5. Optimization of Experimental Conditions

**Figure 4:**
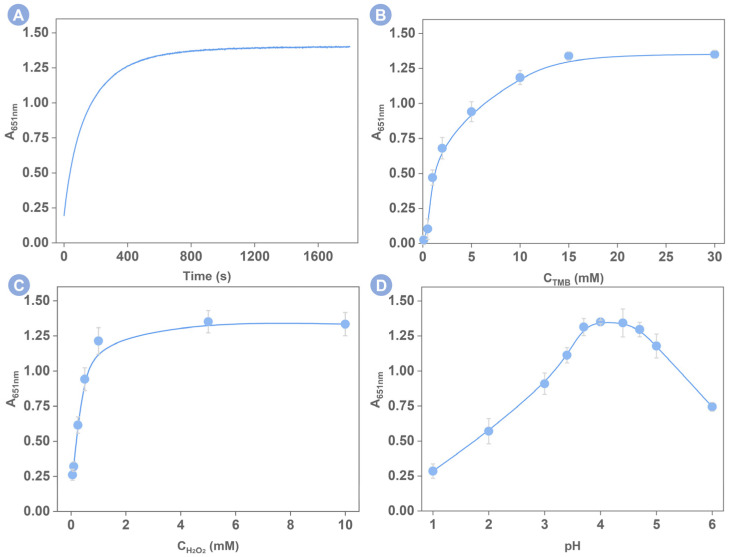
Influences of (**A**) the reaction time, (**B**) the concentration of TMB, (**C**) the concentration of H_2_O_2_, and (**D**) the pH of the reaction solution on the catalytic reaction between TMB and H_2_O_2_. Error bars were obtained from three repetitive experiments.

To achieve the best assay performance, the experimental conditions that are closely related to the catalytical reaction such as the reaction time, the TMB concentration, the H_2_O_2_ concentration, and the pH of the reaction solution, were investigated in Figure 4. The dynamic monitoring of the peak absorption as a function of the reaction time is shown in Figure 4A. The peak absorption was initially increased and then leveled off indicating the optimal reaction time is 10 min. Likewise, when the TMB concentration in Figure 4B was greater than 15 mM and the H_2_O_2_ concentration was greater than 1 M in Figure 4C, the peak absorption also reached a plateau, suggesting the concentration of TMB at 15 mM and the H_2_O_2_ at 1 M are optimal for building the sensing system. Last but not least, we optimized the pH of the reaction solution. As explored in Figure 4D, the employing of 0.1 M acetic acid/sodium acetate buffer to control the pH value of the reaction solution as 4 can achieve the highest signal output. Below or above this value, the peak absorption has deteriorated. We thus controlled the pH value of the reaction solution as 4 to investigate the Hx detection.

### 3.6. Investigation for Quantitative Detection of Hx

**Figure 5:**
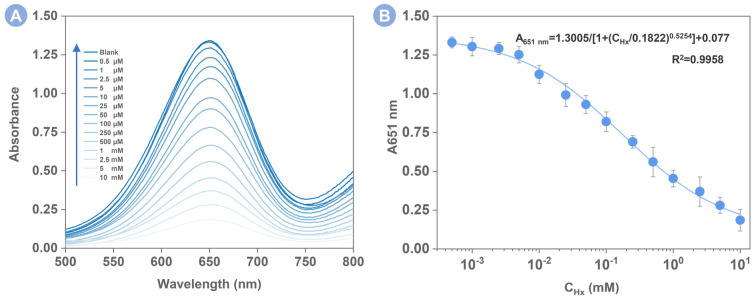
(**A**) Typical UV–vis spectra collected from the PVP-PtNC-based sensing system introduced with Hx at a concentration range from top to bottom: 0, 0.5 μM, 1.0 μM, 2.5 μM, 5.0 μM, 10 μM, 25 μM, 50 μM, 100 μM, 250 μM, 500 μM, 1 mM, 2.5 mM, 5 mM, 1 mM, and 10 mM. (**B**) The dynamic relationship of the peak absorption intensity against the Hx concentration. Error bars were obtained from three repetitive experiments.

Under optimized conditions (Figure 4), the developed colorimetric sensor was used to detect Hx based on the reverse regulation of peroxidase activity of PVP-PtNC. As shown in Figure 5A, with the increase in the Hx concentrations, the UV–vis absorption spectra gradually decreased. This is reasoned by the inhibiting effect of Hx on the PVP-PtNC catalytic activity. Figure 5B plotted the peak absorption at 651 nm dependent on the Hx concentration. In the Hx concentration range from 0 to 10 mM, the four-parameters logistic relationship was obtained between the peak absorption value (A_651_) and the concentration of the Hx. The equation was y = 1.3005/[1 + (x/0.1822)^0.5254^] + 0.077 (R^2^ = 0.9958), where y was the absorbance intensity at 651 nm, and x was the Hx concentration. The limit of detection (LOD) calculated by 3σ/slope is 0.16 μM. Compared with reported fluorescent and electrochemical detection methods or paper-based device in Table S2, the current colorimetric Hx detection method not only shows a lower LOD but also is easy to operate and able to output detectable signals quickly. A previous study has related the concentration of HX to freshness, suggesting that the fish is fresh if the HX concentration is below 250 μM and spoiled at >630 μM [34]. As shown in Figure S3, the colorimetric images show that fresh fish and rotten fish have distinct color differences. Therefore, the colorimetric signal output pattern does not require the use of expensive instruments and professional operators, which can realize semi-quantitative detection directly based on the naked eye.

### 3.7. Investigation for the Qualitative Detection of Hx

**Figure 6:**
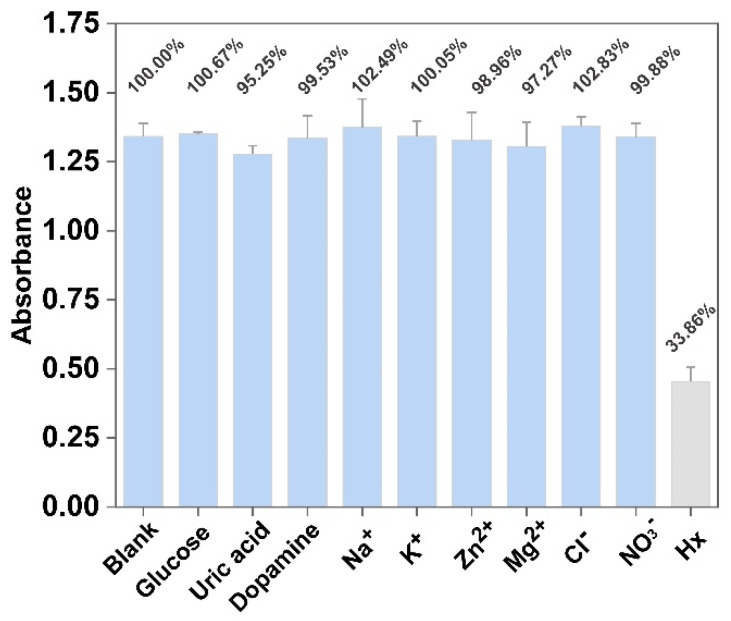
Peak absorption values of the sensing system in the absence and presence of glucose, uric acid, dopamine, Na^+^, K^+^, Zn^2+^, Mg^2+^, Cl^−^, NO_3_^−^, and Hx, respectively. The concentration of Hx was 1 mM, while other non-target species is 5 mM. Error bars were obtained from three repetitive experiments.

As well as the quantitative detection of Hx, the qualitative detection of Hx is another vital point for evaluating the assay ability of this method [35]. To examine this specificity, non-target analytes including glucose, uric acid, dopamine, Na^+^, K^+^, Zn^2+^, Mg^2+^, Cl^−^, and NO_3_^−^ were selected and used to challenge this system. It is worth considering that the metal ions were selected as analytes because some reports have found that the catalysis activity of PVP-PtNC can also be influenced by some metal ions. These metal ions may also exist in fish. These results gathered in Figure 6 show that compared with the negative sample having a high signal absorption, the PVP-PtNC-based sensing system is only Hx responsive considering the significant decrease in the signal. However, the presence of any other non-target analytes, even with a concentration five times higher than Hx, cannot induce any downregulation of the UV–vis signal. The comparative responses demonstrate the excellent specificity of our nanosensing method for the qualitative detection of Hx.

### 3.8. Practicability Investigation

After demonstrating the ability for the quantitative and qualitative detection of Hx, we finally applied the PVP-PtNC-based sensing method to detect Hx from real fish samples, which were prepared by spiking standard Hx at different concentrations into two batches of spoiled fish meat. The pretreat of the fish meat is mentioned above. As shown in Table 1, no matter if the fish samples spoiled for 12 h or 24 h, the spiked Hx at indicated concentrations of 10, 100, and 1000 μM all exhibited favorable recovery rates, which were in the range of 102.6–106.0% and 100.9–101.9%, respectively. Meanwhile, the relative standard derivation (RSD) values were also acceptable. These results affirmed the application potential of this method to detect the amount of Hx in fish meat for monitoring fish freshness.

## 4. Conclusions

In summary, we developed a colorimetric method for the ultrafast signaling of Hx based on the target-triggered reverse regulation of peroxidase activity of PVP-PtNC without the assistance of a natural enzyme. This work shows that the colorimetric method for Hx detection has the following advantages: first, Hx was used to directly regulate the catalytic activity of the nanozyme, which reduced the detection cost and realized the one-step Hx detection with only 10 min, and has been successfully applied to monitor the freshness of live fish; second, the colorimetric method is easy to operate without the assistance of advanced expensive equipment; third, this colorimetric method can detect Hx with high sensitivity and specificity in a wide detection range. We expect this method to have a good application prospect in the freshness detection of fish samples for ensuring food safety.

## Figures and Tables

**Table 1:** Recoveries of the Hx in real fish samples.

Sample	12 h			24 h		
	Spiking (μM)	Found (μM)	Recovery (%)	Spiking (μM)	Found (μM)	Recovery (%)
1	10	10.6	106.0	10	10.15	101.5
2	100	103.5	103.5	100	101.9	101.9
3	1000	1025.6	102.6	1000	1009.7	100.9

## Data Availability

The data are available from the corresponding author.

## Supplementary Materials

The following are available online at https://www.mdpi.com/article/10.3390/foods12020291/s1, Figure S1: (A) TEM image of PVP-PtNCs to show its uniformity and cube shape, (B) Image of single PVP-PtNC taken by a high-resolution TEM, Figure S2: The kinetic assays of PVP-PtNC as artificial peroxidase-like catalysts for oxidation of TMB by H_2_O_2_. (A) Graphic representation of the plots of initial rate (ν) vs. TMB concentration; (B) double-reciprocal plot generated from (A); (C) Graphic representation of ν vs. H_2_O_2_ concentration; (D) double-reciprocal plot generated from (C), Figure S3: The colorimetric images of as-proposed method under different concentrations of Hx, Table S1: Comparison of PVP-PtNC kinetic parameters and HRP, Table S2: Comparison of reported methods with the current one for Hx detection. References [17,29,36,37,38,39,40] are cited in the supplementary materials.
