# Supplementary material for: Downregulation of Peroxidase Activity of Platinum Cube Enables Minute–Time Scale Colorimetric Signaling of Hypoxanthine for Fish Freshness Monitoring
Source: Foods. 2023 Jan 8;12(2):291. doi: 10.3390/foods12020291 (PMC9858192; doi:10.3390/foods12020291)
Supplement: Supplementary file 1 [file foods-12-00291-s001.zip › foods-2110514-supplementary.pdf]

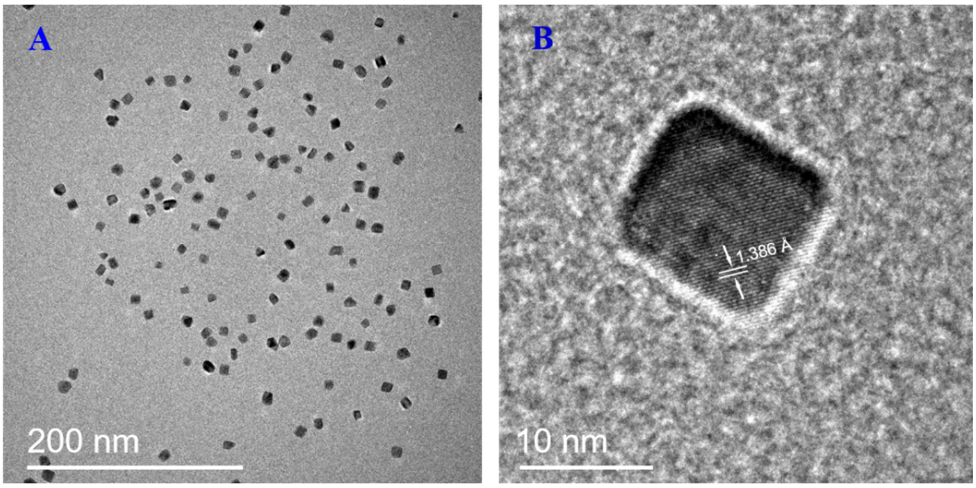

Figure S1. (A) TEM image of PVP-PtNCs to show its uniformity and cube shape, (B) Image of single PVP-PtNC taken by a high-resolution TEM

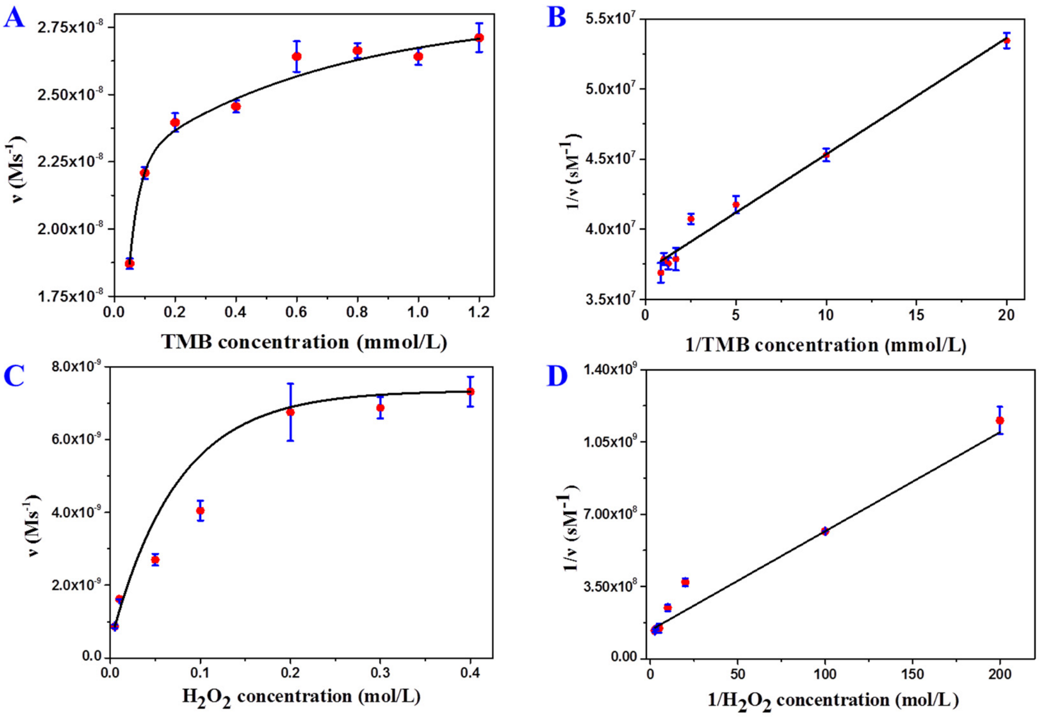

Figure S2. The kinetic assays of PVP-PtNC as artificial peroxidase-like catalysts for oxidation of TMB by H_2O_2. (A) Graphic representation of the plots of initial rate (*v*) vs. TMB concentration; (B) double-reciprocal plot generated from (A); (C) Graphic representation of *v* vs. H_2O_2 concentration; (D) double-reciprocal plot generated from (C)

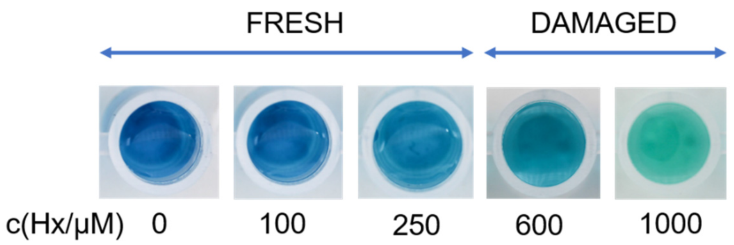

Figure S3. The colorimetric images of as-proposed method under different concentrations of Hx.

Table S1 Comparison of PVP-PtNC kinetic parameters and HRP

Catalyst	[E] (M)[a]	Substrate	V_{max} (Ms^{-1})[b]	K_m(M)[c]	K_{cat} (s^{-1})[d]
PVP-PtNC	1.67×10^{-11}	TMB	1.05×10^{-6}	2.22×10^{-5}	6.29×10^{4}
PVP-PtNC	1.67×10^{-11}	H_2O_2	3.61×10^{-7}	3.92×10^{-3}	2.16×10^{4}
HRP[1]	2.5×10^{-11}	TMB	10.00×10^{-8}	4.34×10^{-4}	4.00×10^{3}
HRP[1]	2.5×10^{-11}	H_2O_2	8.71×10^{-8}	3.70×10^{-3}	3.48×10^{3}

[a][E] is the enzyme (or PVP-PtNC) concentration

[b]*K*m is the Michaelis constant

[c]Vmax is the maximal reaction velocity

[d]*K*cat is the catalytic constant, where *K*cat=Vmax/[E].

Table S2 Comparison of reported methods with the current one for Hx detection

Method	Linear range (μM)	LOD (μM)	Time (min)	Reference
Fluorescent assay	5-120	1.2	15	[2]
Fluorescent assay	8-2500	2.88	60	[3]
Paper-based device	10-700	3.1	30	[4]
Paper-based device	2-10	0.65	30	[5]
Electrochemical assay	20-512	9.5	＞60	[6]
Electrochemical assay	5-60	2	＞60	[7]
Colorimetric assay	0.5-10000	0.16	10	This work

References

1. Gao L, Zhuang J, Nie L, Zhang J, Zhang Y, Gu N, et al. Intrinsic peroxidase-like activity of ferromagnetic nanoparticles. Nat Nanotechnol. 2007;2(9):577-83.
2. Zhang Z, Kwok RTK, Yu Y, Tang BZ, Ng KM. Aggregation-induced emission luminogen-based fluorescence detection of hypoxanthine: a probe for biomedical diagnosis of energy metabolism-related conditions. J Mater Chem B. 2018;6(28):4575-8.
3. Chen J, Lu Y, Yan F, Wu Y, Huang D, Weng Z. A fluorescent biosensor based on catalytic activity of platinum nanoparticles for freshness evaluation of aquatic products. Food Chem. 2020;310:125922.
4. Mustafa F, Andreescu S. Paper-Based Enzyme Biosensor for One-Step Detection of Hypoxanthine in Fresh and Degraded Fish. ACS Sens. 2020;5(12):4092-100.
5. Chen P-C, Li Y-C, Ma J-Y, Huang J-Y, Chen C-F, Chang H-T. Size-tunable copper nanocluster aggregates and their application in hydrogen sulfide sensing on paper-based devices. Sci Rep. 2016;6(1):1-9.
6. Albelda JAV, Uzunoglu A, Santos GNC, Stanciu LA. Graphene-titanium dioxide nanocomposite based hypoxanthine sensor for assessment of meat freshness. Biosens Bioelectron. 2017;89(Pt 1):518-24.
7. Liao L, Xing Y, Xiong X, Gan L, Hu L, Zhao F, et al. An electrochemical biosensor for hypoxanthine detection in vitreous humor: A potential tool for estimating the post-mortem interval in forensic cases. Microchem J. 2020;155:104760.
